# The case for broader use of HOMA-IR in pediatric metabolic assessment: a narrative review

**DOI:** 10.3389/fped.2026.1883916

**Published:** 2026-08-28

**Authors:** Salah Snouda

**Affiliations:** Tunis University, Tunis, Tunisia

**Keywords:** childhood metabolic syndrome, HOMA-IR, insulin resistance indices, metabolic screening, pediatric insulin resistance, precision pediatric medicine, pubertal insulin resistance, targeted screening

## Abstract

The reliance on Body Mass Index (BMI) and fasting glucose as primary indicators of pediatric metabolic health may systematically fail to detect the earliest phase of metabolic dysfunction: chronic hyperinsulinemia. This narrative review, conducted in accordance with the Scale for the Assessment of Narrative Review Articles (SANRA) framework, synthesises current literature to evaluate the evidence for the Homeostatic Model Assessment for Insulin Resistance (HOMA-IR) as a potential first-line tool in pediatric metabolic assessment. We examine the physiological mechanisms of early-onset insulin resistance, the epigenetic and intergenerational consequences of obesogenic environments, the limitations of current diagnostic criteria, and the economic implications of delayed detection. We discuss both universal and targeted screening strategies, comparing HOMA-IR with alternative insulin resistance indices including the Quantitative Insulin Sensitivity Check Index (QUICKI), the Triglyceride-Glucose (TyG) index, and the Single Point Insulin Sensitivity Estimator (SPISE). We critically appraise the principal limitations of HOMA-IR—including insulin assay variability, pubertal physiological variation, and the absence of prospective screening trial data demonstrating improved clinical outcomes. We conclude that while the mechanistic and epidemiological evidence supporting HOMA-IR is substantial, prospective population-level screening trials are required to confirm that HOMA-IR-guided intervention improves clinical outcomes at scale, and we identify such trials as a research priority.

## 1 Introduction

### 1.1 Review methodology

This article is a narrative review conducted in accordance with the SANRA (Scale for the Assessment of Narrative Review Articles) framework for structured narrative reviews. A systematic literature search was conducted in PubMed/MEDLINE, Scopus, and Google Scholar using the following search terms, applied individually and in combination: “HOMA-IR children,” “HOMA-IR pediatric,” “insulin resistance screening children,” “pediatric metabolic syndrome HOMA-IR,” “fasting insulin reference values children,” “childhood insulin resistance cutoff,” “HOMA-IR pubertal,” “pediatric type 2 diabetes screening,” and “HOMA-IR vs. HbA1c children.”

The search was not restricted by publication date but was weighted toward studies published from 2010 onwards to reflect the contemporary evidence base. Studies were included if they: (1) reported HOMA-IR values or cutoffs in pediatric populations (age ≤18 years); (2) compared HOMA-IR with at least one alternative metabolic screening tool; (3) reported outcomes related to metabolic syndrome, insulin resistance, Non-Alcoholic Fatty Liver Disease (NAFLD), or type 2 diabetes (T2D) risk in children; or (4) provided normative reference data for HOMA-IR or related indices in pediatric populations. Studies were excluded if they were case reports, conference abstracts without full-text availability, or studies conducted exclusively in adult populations without pediatric subgroup data. In addition to the primary literature search, reference lists of identified systematic reviews and meta-analyses were hand-searched to identify additional relevant studies. Clinical practice guidelines from the American Diabetes Association (ADA), the International Society for Pediatric and Adolescent Diabetes (ISPAD), the American Academy of Pediatrics (AAP), and the National Institute for Health and Care Excellence (NICE) were reviewed to contextualise the current standard-of-care recommendations.

The narrative synthesis was structured around six thematic domains: (1) the pathophysiology of early-onset insulin resistance; (2) the evidence base for HOMA-IR as a screening tool; (3) the limitations and controversies surrounding HOMA-IR; (4) comparison with alternative insulin resistance indices; (5) the economic and public health implications of delayed detection; and (6) the therapeutic and implementation implications of early HOMA-IR identification. Where the evidence is primarily mechanistic or derived from modelling studies, this is explicitly noted and distinguished from evidence derived from prospective clinical trials.

### 1.2 The current screening landscape

The current pediatric screening model relies heavily on BMI and fasting glucose. However, these markers have well-documented limitations. BMI cannot distinguish between metabolically healthy lean mass and metabolically adverse visceral adiposity; a child with a “normal” BMI may harbour significant visceral fat, chronic low-grade inflammation, and insulin resistance (2). Fasting glucose becomes abnormal only after prolonged compensatory hyperinsulinemia has exhausted pancreatic beta-cell reserve—it reflects late-stage glycaemic dysregulation rather than early metabolic risk (3).

#### 1.2.1 Critical evidence gap

It must be stated at the outset that, as of the time of writing, no prospective population-level screening trial has demonstrated that routine HOMA-IR screening improves clinical outcomes including type 2 diabetes incidence, Metabolic Dysfunction-Associated Steatotic Liver Disease (MASLD) progression, cardiovascular events, quality of life, or long-term mortality. The arguments presented in this review are therefore positioned as an evidence-based rationale for such trials, not as a claim that current evidence is sufficient to mandate universal adoption. This limitation directly affects the strength of the recommendations that follow and should be borne in mind throughout.

### 1.3 Universal versus targeted screening: a balanced perspective

A central question for clinical implementation is whether HOMA-IR should be incorporated into universal screening of all children or targeted screening of high-risk populations. Current pediatric practice generally focuses metabolic assessment on children with established risk factors (19), including:

- Obesity (BMI ≥95th percentile) or severe overweight (BMI 85th–95th percentile)
- Acanthosis nigricans
- Family history of type 2 diabetes (first- or second-degree relative)
- Polycystic Ovary Syndrome (PCOS) or clinical hyperandrogenism
- MASLD/NAFLD
- Small for gestational age (SGA) birth history
- Chronic glucocorticoid exposure
- Selected genetic syndromes associated with insulin resistance (e.g., Turner syndrome, Prader-Willi syndrome)

A targeted screening strategy—incorporating HOMA-IR into the metabolic assessment of children with one or more of these risk factors—represents a pragmatic, lower-cost first step that could be implemented within existing clinical workflows without requiring new infrastructure. This approach would identify the majority of children with clinically significant insulin resistance while avoiding the challenges of universal screening, including the potential for over-diagnosis in low-risk populations and the downstream resource implications of false-positive results.

However, a targeted-only approach has limitations. The concept of the “metabolically obese, normal-weight” child—in whom visceral adiposity and insulin resistance exist despite a normal BMI (2)—suggests that risk-factor-based screening will inevitably miss some affected individuals. Whether the prevalence of insulin resistance in the general pediatric population is sufficient to justify universal screening remains an empirical question that prospective studies must address.

This review presents the evidence supporting HOMA-IR as a screening tool without advocating definitively for either universal or targeted implementation. The optimal strategy will depend on population-specific prevalence data, healthcare system resources, and—most critically—the results of prospective screening trials demonstrating clinical benefit.

## 2 The hyperinsulinemia cascade in the developing body

### 2.1 Pathophysiology of early-onset insulin resistance

The first detectable sign of metabolic dysfunction is not elevated blood sugar; it is elevated fasting insulin. In a child consuming a diet high in ultra-processed foods and refined carbohydrates, repeated postprandial glucose excursions stimulate sustained pancreatic insulin secretion (4). Over time, peripheral tissues (primarily skeletal muscle and liver) downregulate insulin receptor sensitivity, creating a state of insulin resistance (1). The pancreas compensates by increasing insulin output, establishing a cycle of chronic hyperinsulinemia.

This state of chronic hyperinsulinemia is particularly consequential in a developing body, where metabolic infrastructure is still maturing. The hyperinsulinemia cascade may drive several pathological processes simultaneously: hepatic *de novo* lipogenesis, potentially leading to NAFLD or MASLD, which may progress to steatohepatitis (NASH) (5); adipose tissue dysfunction, with expansion beyond physiological capacity and release of pro-inflammatory cytokines (TNF-α, IL-6) that may promote systemic inflammation (6); hormonal disruption, in which chronic insulin elevation may interfere with the hypothalamic-pituitar*y* axis, potentially contributing to early-onset PCOS in girls and altered pubertal timing in boys (7); and neuroinflammation, in which systemic inflammation may cross the blood-brain barrier, potentially contributing to behavioural and cognitive effects (8).

Crucially, longitudinal studies demonstrate that insulin resistance begins to rise in mid-childhood, several years before the onset of puberty (9). This pre-pubertal rise highlights a potential window where intervention could prevent the compounding effects of pubertal insulin resistance.

### 2.2 The natural history of metabolic dysfunction

**Figure 1:**
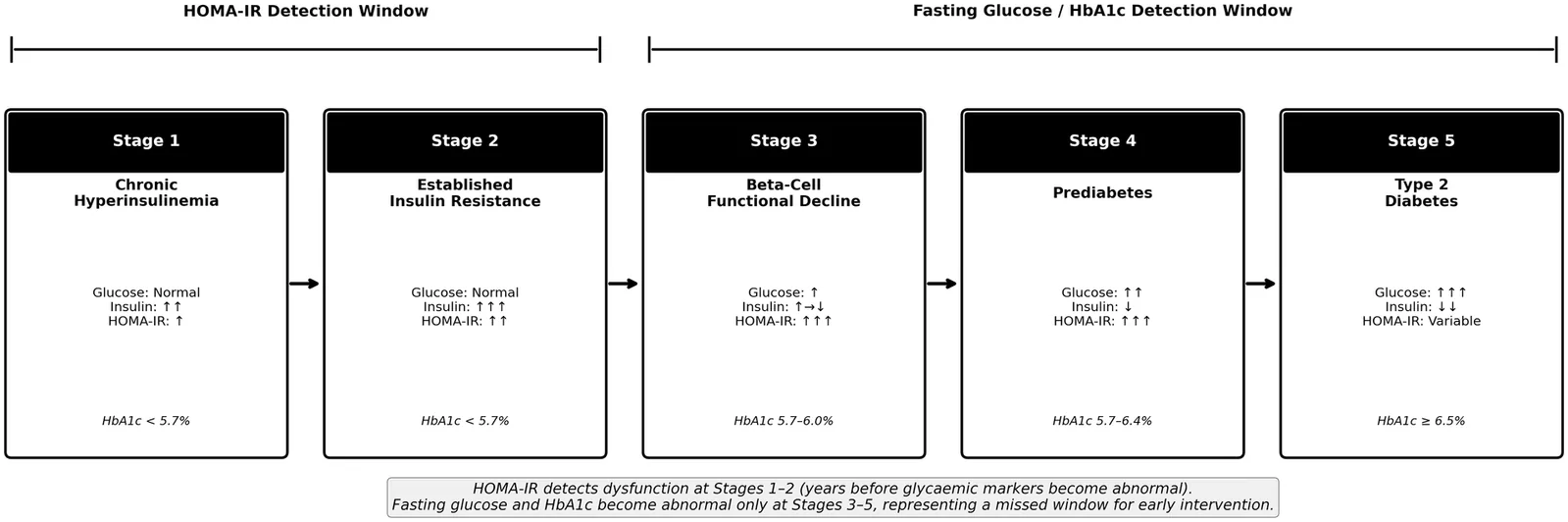
Natural history of pediatric metabolic dysfunction: from compensated hyperinsulinemia to type 2 diabetes. The figure illustrates the five progressive stages of metabolic dysfunction, from initial chronic hyperinsulinemia (Stage 1) through established insulin resistance (Stage 2), beta-cell functional decline (Stage 3), prediabetes (Stage 4), and overt type 2 diabetes (Stage 5). HOMA-IR is positioned to detect dysfunction at Stages 1–2, whereas fasting glucose and HbA1c become abnormal only at Stages 3–5. This temporal advantage represents the principal clinical rationale for HOMA-IR screening.

Figure 1 (proposed) illustrates the progressive natural history of metabolic dysfunction from initial hyperinsulinemia through to established type 2 diabetes:

**Stage 1:** Chronic Hyperinsulinemia (normal glucose, elevated fasting insulin) → **Stage 2:** Established Insulin Resistance (elevated HOMA-IR, compensated glycaemia) → **Stage 3:** Beta-cell Functional Decline (impaired fasting glucose, impaired glucose tolerance) → **Stage 4:** Prediabetes (HbA1c 5.7%–6.4%) → **Stage 5:** Type 2 Diabetes (HbA1c ≥ 6.5%)

HOMA-IR is positioned to detect dysfunction at Stages 1–2, whereas fasting glucose and HbA1c become abnormal only at Stages 3–5. This temporal advantage represents the principal clinical rationale for HOMA-IR screening.

## 3 Epigenetic programming and intergenerational risk

Emerging evidence demonstrates that early-life metabolic environments may alter DNA methylation and histone modification patterns in ways that are transmitted across generations (10–12). The primary mechanism involves insulin-driven alterations to the methylation of genes regulating adipogenesis, hepatic lipid metabolism, and pancreatic beta-cell development. Animal models have demonstrated that offspring of hyperinsulinemic mothers exhibit altered expression of key metabolic genes from birth, independent of postnatal dietary exposure (11). Human cohort data, while more limited, suggest that children born to mothers with gestational insulin resistance exhibit higher HOMA-IR values in early childhood than children born to metabolically healthy mothers, even after controlling for postnatal diet and BMI (12).

The clinical implication is that HOMA-IR screening in children may identify not only those at individual risk, but those whose risk is epigenetically amplified by maternal metabolic history—a population that may require earlier and more intensive monitoring. However, it should be noted that the epigenetic evidence is primarily derived from animal models and observational human cohorts; direct causal evidence in human populations remains limited.

## 4 HOMA-IR: evidence base and clinical application

### 4.1 The HOMA-IR formula

The Homeostatic Model Assessment for Insulin Resistance (HOMA-IR) is calculated as follows:

**Using mg/dL units:** >HOMA-IR = (Fasting Insulin [µIU/mL] × Fasting Glucose [mg/dL])/405.

**Using mmol/L units:** >HOMA-IR = [Fasting Insulin (µIU/mL) × Fasting Glucose (mmol/L)]/22.5.

HOMA-IR quantifies the degree of basal (primarily hepatic) insulin resistance from a single fasting blood sample (13). While the hyperinsulinemic-euglycaemic clamp remains the gold standard for measuring insulin sensitivity, it is highly invasive and impractical for clinical screening. HOMA-IR provides a practical surrogate of basal insulin resistance (13), but it is not interchangeable with the hyperinsulinemic-euglycaemic clamp and requires age-, pubertal-stage-, population-, and assay-specific interpretation (15–20, 26, 28).

### 4.2 Establishing pediatric reference ranges

The scientific literature supports the utility of HOMA-IR in children, though establishing universal cutoffs has been challenging due to physiological changes during puberty. Consensus from multiple large-scale cohorts suggests that values >2.5 consistently correlate with increased metabolic risk in prepubertal children (15, 16). However, these values must be interpreted with the following caveats:

**Important interpretive considerations:**

1. **Age dependence:** HOMA-IR values increase physiologically during puberty and decline in late adolescence.
2. **Pubertal stage:** Values during Tanner stages II–IV are physiologically higher than prepubertal or post-pubertal values.
3. **Sex differences:** Girls typically exhibit higher HOMA-IR values during puberty than boys of the same age.
4. **Ethnicity:** Population-specific reference ranges are required; cutoffs derived from European Caucasian populations may not apply to other ethnic groups (28).
5. **Insulin assay methodology:** Different immunoassays can yield insulin values differing by 20%–40% from the same sample (26).
6. **Laboratory calibration:** Results from different laboratories are not directly comparable.

The application of age-, sex-, and pubertal-stage-specific reference ranges is therefore essential for responsible clinical use. The large-scale study by Hammel et al. (31) provides age-stratified normative data from early childhood through adulthood (31), and recent data from Acta Paediatrica provide practical clinical thresholds for children with excess weight stratified by age, sex, and pubertal stage (32).

It must be explicitly acknowledged that, as of the time of writing, no major international pediatric or endocrine society—including the AAP, ISPAD, or the Pediatric Endocrine Society (PES)—has issued a formal recommendation for the routine clinical use of HOMA-IR as a standardised screening tool in children. This absence of formal endorsement reflects the evidence gap that this review seeks to highlight and that prospective screening trials must address.

### 4.3 Comparative screening performance

To contextualise the potential role of HOMA-IR within the broader screening landscape, Table 1 compares the principal metabolic screening tools currently available or under investigation in pediatric populations:

**Table 1:** Comparison of pediatric metabolic screening approaches.

Screening tool	Pathophysiological domain	Stage of dysfunction detected	Clinical feasibility	Cost	Pediatric evidence base
HOMA-IR	Basal hepatic insulin resistance	Stage 1—Hyperinsulinemia (pre-dysglycemia)	High—fasting blood draw only	Variable by laboratory and healthcare system	Strong—multiple large-scale cohorts (15–18)
Fasting Glucose	Beta-cell failure/glycaemic dysregulation	Stage 3—Established dysglycemia	High	Very low	Established but late-stage
HbA1c	Chronic glycaemic exposure	Stage 3—Established dysglycemia	High	Low	Established but late-stage
BMI	Total body mass (not metabolic)	Does not detect metabolic dysfunction	Very high	Negligible	Poor—misses metabolically obese normal-weight children
TyG Index	Hepatic insulin resistance (dyslipidemia)	Stage 2—Hepatic lipid overflow	High—fasting glucose + triglycerides	Low	Growing—several pediatric studies (29, 30)
Oral GTT	Dynamic glucose handling	Stage 2–3	Moderate—2-hour test required	Moderate	Strong but impractical for universal screening
CGM	Dynamic glucose variability	Stage 2–3	Low—device required	High	Emerging—not yet validated for universal pediatric screening

BMI, body mass index; CGM, continuous glucose monitoring; GTT, glucose tolerance test; HbA1c, glycated haemoglobin; HOMA-IR, Homeostatic Model Assessment for Insulin Resistance; TyG, Triglyceride-Glucose index.

### 4.4 Comparison with alternative insulin resistance indices

Beyond the broader screening tools compared in Table 1, HOMA-IR must also be evaluated against other validated surrogate indices specifically designed to estimate insulin sensitivity (Table 2).

**Table 2:** Comparison of surrogate insulin resistance indices applicable to pediatric populations.

Index	Formula/Derivation	What It measures	Advantages	Limitations	Pediatric evidence
HOMA-IR	(FI × FG)/405	Basal hepatic IR	Simple, single fasting sample, extensive pediatric data	Assay-dependent, static measure	Strong—multiple large cohorts (15–18)
QUICKI	1/(log FI + log FG)	Basal insulin sensitivity	Mathematically equivalent to HOMA-IR; better distribution	Same limitations as HOMA-IR	Moderate—validated in children (33)
TyG Index	Ln(TG × FG/2)	Hepatic lipid-driven IR	No insulin assay needed	Detects Stage 2 (lipid overflow), not Stage 1	Growing—several pediatric studies (29, 30)
SPISE	600 × HDL^0.185/(TG^0.2 × BMI^1.338)	Whole-body insulin sensitivity (surrogate)	No fasting insulin measurement needed	Requires a lipid panel; limited validation across pediatric populations	Initial validation included white juveniles and adults; broader pediatric validation is needed (34)
Matsuda Index	Derived from OGTT	Whole-body insulin sensitivity	Captures dynamic glucose handling	Requires 2-hour OGTT; impractical for screening	Strong but impractical for universal use
Insulinogenic Index	*Δ*Insulin₃₀/*Δ*Glucose₃₀	Beta-cell secretory response	Assesses beta-cell function	Requires OGTT	Limited pediatric screening data
Disposition Index	Insulinogenic Index × Matsuda	Beta-cell compensation for IR	Integrates secretion and sensitivity	Requires OGTT	Research tool; not screening-applicable

BMI, body mass index; FG, fasting glucose; FI, fasting insulin; HDL, high-density lipoprotein cholesterol; HOMA-IR, Homeostatic Model Assessment for Insulin Resistance; IR, insulin resistance; OGTT, oral glucose tolerance test; QUICKI, Quantitative Insulin Sensitivity Check Index; SPISE, Single Point Insulin Sensitivity Estimator; TG, triglycerides; TyG, Triglyceride-Glucose index.

Compared with HOMA-IR, the TyG index has been investigated as a potential pediatric surrogate of insulin resistance because it does not require a fasting insulin measurement (29, 30). Some studies suggest TyG may have comparable or even superior predictive value for metabolic syndrome in specific pediatric cohorts (30). However, TyG measures a downstream consequence of insulin resistance (hepatic lipid overflow manifesting as hypertriglyceridaemia) rather than the primary pathophysiological process (hyperinsulinemia). A child may exhibit clinically significant hyperinsulinemia (detectable by HOMA-IR) before triglyceride elevation occurs (detectable by TyG). This temporal difference suggests that HOMA-IR and TyG may be complementary rather than competing tools, with HOMA-IR detecting earlier-stage dysfunction.

The SPISE index, validated in cohorts including white juveniles and adults without diabetes (34), offers the advantage of not requiring fasting insulin measurement; broader validation across pediatric populations is still needed.

### 4.5 HOMA-IR in the context of precision pediatric medicine

The emerging precision diabetology framework, as articulated by the ADA/EASD Consensus Report on Precision Medicine in Diabetes, emphasises that metabolic disease encompasses biologically distinct subtypes requiring individualised approaches (35). This raises an important question: is a single fasting-state surrogate sufficient to capture the full complexity of pediatric insulin resistance?

We acknowledge that HOMA-IR, as a basal hepatic insulin resistance surrogate, does not fully characterise the multidimensional nature of the insulin resistance syndrome, which also encompasses peripheral (skeletal muscle) insulin resistance, adipose tissue dysfunction, and dynamic postprandial glucose handling. More sophisticated phenotyping tools—including oral glucose tolerance testing and emerging multi-omic approaches—may offer a more complete biological picture.

However, the practical reality of pediatric primary care demands a pragmatic first-line approach. HOMA-IR is positioned in this review not as a comprehensive phenotyping instrument, but as a potential clinical gateway to more detailed metabolic assessment—the tool that identifies which children may require more sophisticated evaluation. This is analogous to the role of HbA1c in adult T2D screening: it is not a complete characterisation of the metabolic phenotype, but it may serve as an initial step that determines who enters the precision medicine pathway.

## 5 Limitations and controversies: a critical assessment

While the utility of HOMA-IR in pediatric metabolic assessment is increasingly recognised, its universal adoption has been hindered by several substantive limitations. Addressing these honestly is essential for responsible clinical implementation.

### 5.1 Insulin assay variability

The most significant barrier to establishing universal HOMA-IR cutoffs is the variability in fasting insulin measurement across different laboratories (20). Different immunoassays can yield insulin values that differ by 20%–40% from the same blood sample (26), meaning that a HOMA-IR value calculated from one laboratory's assay cannot be directly equated to a value from another.

#### 5.1.1 Practical clinical implications

This variability means that:—Clinicians should utilise the same laboratory for serial measurements within an individual patient, prioritising the trajectory of HOMA-IR over any single absolute value.—Comparisons against published numerical thresholds (e.g., >2.5 or >3.5) must be interpreted with caution unless the threshold was derived using the same assay methodology.—Laboratory-specific reference ranges, ideally derived from local healthy pediatric populations, are more informative than universal numerical cutoffs.—Serial measurements performed within the same laboratory are considerably more clinically informative than single-point comparisons against universal thresholds.

The International Federation of Clinical Chemistry and Laboratory Medicine (IFCC), in collaboration with the ADA, maintains an active Working Group on Standardisation of Insulin Assays (WG-SIA) aiming to harmonise assays to a uniform reference system (27). Until universal standardisation is achieved, the clinical utility of HOMA-IR is best realised through within-patient longitudinal monitoring rather than rigid categorical classification.

### 5.2 Pubertal physiological insulin resistance

Puberty induces a state of transient, physiological insulin resistance that is essential to understand when interpreting HOMA-IR in pediatric populations. The following physiological considerations are critical:

#### 5.2.1 Growth hormone-mediated insulin resistance

The primary driver of pubertal insulin resistance is the surge in growth hormone (GH) and insulin-like growth factor 1 (IGF-1) secretion during puberty. GH directly antagonises insulin signalling in peripheral tissues, reducing glucose uptake in skeletal muscle and increasing hepatic glucose output. This is a physiological adaptation that supports the anabolic demands of pubertal growth (14).

#### 5.2.2 Sex differences

Girls typically develop higher HOMA-IR values during puberty than boys, reflecting greater physiological insulin resistance during female pubertal development. This sex difference peaks during mid-puberty (Tanner stages III–IV) and narrows in late adolescence (14).

#### 5.2.3 Timing across tanner stages

Insulin sensitivity decreases by approximately 25%–50% during the pubertal transition, with the nadir occurring at Tanner stages II–III. The degree of physiological insulin resistance varies substantially between individuals (9, 14).

#### 5.2.4 Interaction with obesity

In children with pre-existing obesity, pubertal insulin resistance compounds the pathological insulin resistance associated with adiposity, creating a “double hit” that may accelerate progression toward metabolic dysfunction. Distinguishing physiological from pathological insulin resistance in obese pubertal adolescents requires careful clinical judgement and longitudinal monitoring (15).

#### 5.2.5 Spontaneous improvement after puberty

Physiological pubertal insulin resistance typically resolves spontaneously in late adolescence (Tanner stage V) as GH secretion normalises. HOMA-IR values that remain elevated beyond the pubertal transition are more likely to reflect pathological insulin resistance. This temporal pattern underscores the importance of longitudinal monitoring rather than single-point screening during puberty.

The solution is the application of age-, sex-, and pubertal-stage-specific reference ranges. The existence of physiological variation does not invalidate HOMA-IR as a screening tool; rather, it demands clinical nuance and the use of appropriate, stage-specific thresholds. Applying a static prepubertal cutoff (e.g., >2.5) to a pubertal adolescent will inevitably result in over-diagnosis.

### 5.3 Ethnic and racial variation

HOMA-IR cutoffs derived from European Caucasian populations may not accurately reflect metabolic risk in other ethnicities. South Asian populations tend to develop insulin resistance and T2D at lower BMI and potentially lower HOMA-IR thresholds, while some African-American cohorts exhibit higher fasting insulin levels as a normal physiological variant (28). As with growth charts, the clinical application of HOMA-IR requires population-specific reference ranges. Large-scale cohorts are actively defining these percentiles globally, allowing for more precise, ethnically appropriate risk stratification.

### 5.4 Absence of prospective outcome data

The most fundamental limitation of the HOMA-IR screening argument is the absence of prospective evidence demonstrating that routine screening improves clinical outcomes. Specifically, no completed randomised controlled trial has demonstrated that HOMA-IR-guided screening and intervention reduces:

- Incidence of type 2 diabetes
- Progression of MASLD/NAFLD
- Cardiovascular events
- Quality of life
- Long-term mortality

This evidence gap is not unique to HOMA-IR—it applies to all proposed metabolic screening strategies in children—but it directly limits the strength of any recommendation for routine implementation. The arguments in this review are based on mechanistic plausibility and cross-sectional associations, not on completed screening trials; economic modelling specific to HOMA-IR screening is also lacking. Prospective validation remains the most urgent research priority.

## 6 The economic implications of delayed detection

### 6.1 Direct costs of pediatric metabolic disease

The lifetime direct medical costs of treating type 2 diabetes are estimated at over $85,000 per individual, with the majority of costs stemming from the management of complications (21). When the disease begins in childhood, the duration of exposure to hyperglycaemia increases, leading to earlier and more severe microvascular and macrovascular complications, further inflating lifetime costs (22).

An economic simulation of pediatric diabetes and dysglycemia screening compared oral glucose tolerance, glucose-challenge, random glucose, and HbA1c strategies; it did not evaluate HOMA-IR. The cost-effectiveness of adding HOMA-IR to targeted pediatric screening therefore remains unknown (23).

### 6.2 Downstream costs of screening

However, a comprehensive cost-effectiveness assessment must also consider the downstream investigations and clinical activity generated by abnormal HOMA-IR results, including:

- Repeat laboratory assessments (confirmatory fasting insulin, glucose)
- Oral glucose tolerance testing for children with elevated HOMA-IR
- Hepatic imaging (ultrasound) for MASLD assessment
- Inflammatory biomarker panels (hs-CRP, ALT, lipid profile)
- Specialist referrals (paediatric endocrinology, hepatology)
- Follow-up visits and longitudinal monitoring

These downstream costs are difficult to estimate without prospective implementation data. A targeted screening strategy (Section 1.3) would substantially reduce these costs by limiting screening to children with established risk factors. The net cost-effectiveness of HOMA-IR screening—whether universal or targeted—remains an empirical question that implementation studies must address.

### 6.3 The window of reversibility

The principal economic argument for early detection is the concept of reversibility. Physical activity may improve insulin resistance in youth (24). In a small pilot randomised trial involving adolescents with obesity and non-alcoholic fatty liver disease, a carbohydrate-restricted diet reduced hepatic lipid within the intervention group and produced greater reductions in HOMA-IR than a fat-restricted diet; these findings require confirmation in larger trials (25). Once pancreatic beta-cell function declines and T2D is established, achieving remission becomes substantially more difficult. Early identification through HOMA-IR may therefore open a therapeutic window in which low-cost lifestyle interventions can prevent the need for expensive long-term pharmacological management.

It is important to note that direct evidence from completed population-level HOMA-IR screening programmes is limited, and the cost-effectiveness of HOMA-IR screening has not been established.

## 7 From screening to action: therapeutic implications

### 7.1 Lifestyle and nutritional intervention as first-line response

Physical activity and diet quality may improve insulin resistance in youth. In a small randomised study of adolescents with obesity, HOMA-IR decreased following an eight-week lifestyle programme, while supervised exercise improved fitness and power (24). In a separate pilot randomised trial involving adolescents with obesity and non-alcoholic fatty liver disease, a carbohydrate-restricted diet reduced hepatic lipid within the intervention group and produced greater reductions in HOMA-IR than a fat-restricted diet; these findings require confirmation in larger trials (25). These interventions are non-pharmacological, low-cost, and reversible, and may be initiated upon HOMA-IR identification without specialist referral.

### 7.2 Pharmacological considerations

Metformin is the only pharmacological agent currently approved for use in children aged ≥10 years with established T2D in most jurisdictions. Its mechanism—primarily hepatic glucose production suppression and mild insulin sensitisation—is directly relevant to the HOMA-IR phenotype. However, the identification of elevated HOMA-IR before overt T2D onset may create a window in which lifestyle intervention precludes the need for pharmacological therapy entirely, which is the preferred clinical outcome.

SGLT-2 inhibitors, GLP-1 receptor agonists, and dual incretin therapies are not currently approved for pediatric use in most jurisdictions. However, their adult evidence base provides a rationale for future pediatric trials in HOMA-IR-stratified populations. A proposed expert-opinion therapeutic response framework for HOMA-IR-stratified pediatric patients is presented in Table 3.

**Table 3:** Proposed expert-opinion therapeutic response framework for HOMA-IR-stratified pediatric patients.

HOMA-IR level	Proposed risk category	Proposed first-line response	Proposed second-line response	Specialist referral
<2.5 (prepubertal)	Low risk	Routine monitoring (annual)	—	Not indicated
2.5–3.5	Borderline elevated	Dietary counselling; reduce ultra-processed food intake; increase physical activity	Repeat HOMA-IR at 3 months	Consider if no improvement at 6 months
3.5–5.0	Elevated	Intensive dietary intervention; structured exercise programme; family-based lifestyle modification	Hepatic ultrasound; inflammatory biomarker panel	Paediatric endocrinology referral
>5.0	Severely elevated	Immediate specialist referral; consider metformin (age ≥10 with established T2D); oral GTT	Full metabolic panel; NAFLD assessment	Paediatric endocrinology + hepatology

GTT, glucose tolerance test; HOMA-IR, Homeostatic Model Assessment for Insulin Resistance; MASLD, Metabolic Dysfunction-Associated Steatotic Liver Disease; NAFLD, Non-Alcoholic Fatty Liver Disease; T2D, type 2 diabetes.

### 7.3 Proposed therapeutic response framework

#### 7.3.1 Critical interpretive note

The thresholds and management pathways in Table 3 represent the authors' proposed framework based on synthesis of the available literature. They are not validated clinical cut-offs and are not endorsed by any international pediatric or endocrine society. HOMA-IR values vary according to age, pubertal stage, sex, ethnicity, and insulin assay methodology. Clinicians should interpret these values in the context of age-, sex-, and pubertal-stage-specific reference ranges appropriate to their local population and laboratory. The management strategies proposed here should be considered as expert opinion requiring prospective validation, not as evidence-based clinical guidelines.

### 7.4 Proposed validation framework

The clinical utility of HOMA-IR-guided intervention could be validated through a prospective, randomised controlled trial in which children identified as high-risk by HOMA-IR screening are randomised to standard care vs. HOMA-IR-guided intensive lifestyle intervention. Proposed primary endpoints would include HOMA-IR normalisation at 12 months; proposed secondary endpoints would include hepatic steatosis regression on ultrasound, hs-CRP reduction, and T2D incidence at 5-year follow-up. Such a trial would provide the direct clinical outcome evidence that currently represents the most significant gap in the HOMA-IR screening evidence base.

## 8 Clinical feasibility and implementation considerations

### 8.1 Laboratory infrastructure

HOMA-IR requires a fasting blood draw and a reliable insulin immunoassay—both of which are available in standard clinical laboratory settings in most high-income healthcare systems. The calculation itself is simple enough to be automated within any electronic health record system. We acknowledge that insulin assay availability and standardisation remain variable in low- and middle-income countries, and propose that investment in insulin assay infrastructure represents a priority in these settings.

### 8.2 Tiered implementation model

We propose a tiered implementation model for clinical use:

**Tier 1 (Primary Care/Well-Child Visits):** HOMA-IR as a first-line metabolic screen alongside standard blood glucose and lipid assessment. This tier requires no additional infrastructure beyond a fasting blood draw. Initially, this could be targeted to children with established risk factors (Section 1.3).

**Tier 2 (Specialist Referral):** For children with elevated HOMA-IR (>3.5, adjusted for age and pubertal stage), additional assessments may include oral glucose tolerance testing, hepatic ultrasound, and inflammatory biomarker assessment (hs-CRP, ALT). DEXA scanning, salivary cortisol testing, and continuous glucose monitoring are not proposed as first-line screening tools; they are reserved for specialist-level assessment in children with confirmed metabolic risk.

## 9 Conclusion

This narrative review presents the evidence supporting broader use of HOMA-IR in pediatric metabolic assessment. The mechanistic rationale is substantial: HOMA-IR detects chronic hyperinsulinemia—the earliest measurable phase of metabolic dysfunction—years before fasting glucose or HbA1c become abnormal. The epidemiological evidence from multiple large-scale pediatric cohorts consistently demonstrates associations between elevated HOMA-IR and metabolic syndrome, NAFLD, and T2D risk. The tool is inexpensive, requires only a fasting blood sample, and can be integrated into existing clinical workflows.

However, the evidence base has important limitations that must be honestly acknowledged. No prospective population-level screening trial has demonstrated that HOMA-IR-guided screening and intervention improves clinical outcomes. The proposed thresholds are not validated clinical cut-offs. Insulin assay variability complicates cross-laboratory comparisons. Pubertal physiology requires careful interpretive nuance. The optimal screening strategy (universal vs. targeted) remains undetermined.

We therefore conclude that:

1. The mechanistic and epidemiological evidence supports HOMA-IR as a potentially valuable addition to pediatric metabolic assessment, particularly in children with established risk factors.
2. The integration of HOMA-IR into targeted screening of high-risk pediatric populations represents a pragmatic first step that could be implemented within existing clinical infrastructure.
3. The most urgent research priority is the design and funding of prospective, randomised screening trials—with HOMA-IR normalisation, NAFLD regression, and T2D incidence as primary endpoints—to determine whether HOMA-IR-guided intervention improves clinical outcomes at scale.
4. International standardisation of insulin assays remains essential for the responsible clinical application of HOMA-IR across healthcare systems.

The question is not whether HOMA-IR provides earlier detection of metabolic dysfunction than current screening tools—the evidence suggests it does. The question is whether earlier detection translates into improved clinical outcomes, and this can only be answered by prospective trials.

## References

[1] Tagi VM Giannini C Chiarelli F. Insulin resistance in children. Front Endocrinol (Lausanne). (2019) 10:342. 10.3389/fendo.2019.00342 31214120 PMC6558106

[2] Peplies J Jiménez-Pavón D Savva SC Buck C Günther K Fraterman A. Percentiles of fasting serum insulin, glucose, HbA1c and HOMA-IR in pre-pubertal normal weight European children from the IDEFICS cohort. Int J Obes (Lond). (2014) 38(Suppl 2):S39–47. 10.1038/ijo.2014.134 25376219

[3] American Diabetes Association Professional Practice Committee for Diabetes. 14. Children and Adolescents: Standards of Care in Diabetes—2026. Diabetes Care. (2026) 49(Suppl 1):S297–S320. 10.2337/dc26-S014 PMC12690182 41358890

[4] Costa CS Rauber F Leffa PS Sangalli CN Campagnolo PDB Vitolo MR. Ultra-processed food consumption and its effects on anthropometric and glucose profile: a longitudinal study during childhood. Nutr Metab Cardiovasc Dis. (2019) 29(2):177–84. 10.1016/j.numecd.2018.11.003 30660687

[5] Farías C Cisternas C Gana JC Alberti G Echeverría F Videla LA. Dietary and nutritional interventions in nonalcoholic fatty liver disease in pediatrics. Nutrients. (2023) 15(22):4829. 10.3390/nu15224829 PMC10674812 38004223

[6] Calcaterra V Verduci E Vandoni M Rossi V Fiore G Massini G. The effect of healthy lifestyle strategies on the management of insulin resistance in children and adolescents with obesity: a narrative review. Nutrients. (2022) 14(21):4692. 10.3390/nu14214692 36364954 PMC9657567

[7] Thota P Perez-Lopez FR Benites-Zapata VA Pasupuleti V Hernandez AV. Obesity-related insulin resistance in adolescents: a systematic review and meta-analysis of observational studies. Gynecol Endocrinol. (2017) 33(3):179–84. 10.1080/09513590.2016.1273897 28102091

[8] Calcaterra V Cena H Rossi V Santero S Bianchi A Zuccotti G. Ultra-processed food, reward system and childhood obesity. Children. (2023) 10(5):804. 10.3390/children10050804 37238352 PMC10217200

[9] Jeffery AN Metcalf BS Hosking J Streeter AJ Voss LD Wilkin TJ. Age before stage: insulin resistance rises before the onset of puberty: a 9-year longitudinal study (EarlyBird 26). Diabetes Care. (2012) 35(3):536–41. 10.2337/dc11-1281 22279034 PMC3322712

[10] Fernandez-Twinn DS Constância M Ozanne SE. Intergenerational epigenetic inheritance in models of developmental programming of adult disease. Semin Cell Dev Biol. (2015) 43:85–95. 10.1016/j.semcdb.2015.06.006 26135290 PMC5844462

[11] Panera N Mandato C Crudele A Bertrando S Vajro P Alisi A. Genetics, epigenetics and transgenerational transmission of obesity in children. Front Endocrinol (Lausanne). (2022) 13:1006008. 10.3389/fendo.2022.1006008 36452324 PMC9704419

[12] Vickers MH. Developmental programming and transgenerational transmission of obesity. Ann Nutr Metab. (2014) 64(Suppl. 1):26–34. 10.1159/000360506 25059803

[13] Wallace TM Levy JC Matthews DR. Use and abuse of HOMA modeling. Diabetes Care. (2004) 27(6):1487–95. 10.2337/diacare.27.6.1487 15161807

[14] Goran MI Gower BA. Longitudinal study on pubertal insulin resistance. Diabetes. (2001) 50(11):2444–50. 10.2337/diabetes.50.11.2444 11679420

[15] Kurtoğlu S Hatipoğlu N Mazıcıoğlu M Kendirici M Keskin M Kondolot M. Insulin resistance in obese children and adolescents: hOMA-IR cut-off levels in the prepubertal and pubertal periods. J Clin Res Pediatr Endocrinol. (2010) 2(3):100–6. 10.4274/jcrpe.v2i3.100 PMC3005684 21274322

[16] Shashaj B Luciano R Contoli B Morino GS Spreghini MR Rustico C. Reference ranges of HOMA-IR in normal-weight and obese young caucasians. Acta Diabetol. (2016) 53(2):251–60. 10.1007/s00592-015-0782-4 26070771

[17] Arellano-Ruiz P García-Hermoso A Cavero-Redondo I Pozuelo-Carrascosa D Martínez-Vizcaíno V Solera-Martinez M. Homeostasis model assessment cut-off points related to metabolic syndrome in children and adolescents: a systematic review and meta-analysis. Eur J Pediatr. (2019) 178(12):1813–22. 10.1007/s00431-019-03464-y 31522316

[18] Yin J Li M Xu L Wang Y Cheng H Zhao X. Insulin resistance determined by homeostasis model assessment (HOMA) and associations with metabolic syndrome among Chinese children and teenagers. Diabetol Metab Syndr. (2013) 5(1):71. 10.1186/1758-5996-5-71 24228769 PMC3833654

[19] Lim CYS Foo YW Tok CLX Lim YY Loke KY Lee YS. Screening for metabolic complications of childhood and adolescent obesity: a scoping review of national and international guidelines. Obes Rev. (2022) 23(12):e13513. 10.1111/obr.13513 36286080

[20] Schrank Y Fontes R Perozo AFDF Araújo PB Pinheiro MFMC Gomes DMV. Proposal for fasting insulin and HOMA-IR reference intervals based on an extensive Brazilian laboratory database. Arch Endocrinol Metab. (2024) 68:e230483. 10.20945/2359-4292-2023-0483 39529982 PMC11554367

[21] Zhuo X Zhang P Hoerger TJ. Lifetime direct medical costs of treating type 2 diabetes and diabetic complications. Am J Prev Med. (2013) 45(3):253–61. 10.1016/j.amepre.2013.04.017 23953350

[22] Rodriquez IM O’Sullivan KL. Youth-onset type 2 diabetes: burden of complications and socioeconomic cost. Curr Diab Rep. (2023) 23(5):59–67. 10.1007/s11892-023-01501-7 36961664 PMC10037371

[23] Wu EL Kazzi NG Lee JM. Cost-effectiveness of screening strategies for identifying pediatric diabetes mellitus and dysglycemia. JAMA Pediatr. (2013) 167(1):32–9. 10.1001/jamapediatrics.2013.419. 23403824 PMC3837695

[24] McCormack SE McCarthy MA Harrington SG Farilla L Hrovat MI Systrom DM. Effects of exercise and lifestyle modification on fitness, insulin resistance, skeletal muscle oxidative phosphorylation and intramyocellular lipid content in obese children and adolescents. Pediatr Obes. (2014) 9(4):281–91. 10.1111/j.2047-6310.2013.00180.x 23801526 PMC3808470

[25] Goss AM Dowla S Pendergrass M Ashraf A Bolding M Morrison S. Effects of a carbohydrate-restricted diet on hepatic lipid content in adolescents with non-alcoholic fatty liver disease: a pilot, randomized trial. Pediatr Obes. (2020) 15(7):e12630. 10.1111/ijpo.12630. 32128995

[26] Staten MA Stern MP Miller WG Steffes MW Campbell SE. Insulin assay standardization: leading to measures of insulin sensitivity and secretion for practical clinical care. Diabetes Care. (2010) 33(1):205–6. 10.2337/dc09-1206 20040676 PMC2797975

[27] International Federation of Clinical Chemistry and Laboratory Medicine (IFCC). Standardisation of Insulin Assays (WG-SIA) (n.d.). Available online at: https://ifcc.org/ifcc-scientific-division/sd-working-groups/wg-sia/ (Accessed June 15, 2026).

[28] Ehtisham S Crabtree N Clark P Shaw N Barrett T. Ethnic differences in insulin resistance and body composition in United Kingdom adolescents. J Clin Endocrinol Metab. (2005) 90(7):3963–9. 10.1210/jc.2004-2001 15840754

[29] Kang B Yang Y Lee EY Yang HK Kim H-S Lim S-Y. Triglycerides/glucose index is a useful surrogate marker of insulin resistance among adolescents. Int J Obes (Lond). (2017) 41(5):789–92. 10.1038/ijo.2017.14 28104918

[30] Dundar C Terzi O Arslan HN. Comparison of the ability of HOMA-IR, VAI, and TyG indexes to predict metabolic syndrome in children with obesity: a cross-sectional study. BMC Pediatr. (2023) 23(1):74. 10.1186/s12887-023-03892-8 36765298 PMC9921359

[31] Hammel MC Stein R Kratzsch J Vogel M Eckert AJ Triatin RD. Fasting indices of glucose-insulin-metabolism across life span and prediction of glycemic deterioration in children with obesity from new diagnostic cut-offs. Lancet Reg Health Eur. (2023) 30:100652. 10.1016/j.lanepe.2023.100652 37465325 PMC10350850

[32] Sarıkaya E Kilci F Murat NÖ. Fasting biomarkers and glycated haemoglobin identify children with excess weight at risk of dysglycaemia and guide oral glucose tolerance testing. Acta Paediatr. (2026) 115(10):2305–14. 10.1111/apa.70657 PMC13552578 42332988

[33] Katz A Nambi SS Mather K Baron AD Follmann DA Sullivan G. Quantitative insulin sensitivity check Index: a simple, accurate method for assessing insulin sensitivity in humans. J Clin Endocrinol Metab. (2000) 85(7):2402–10. 10.1210/jcem.85.7.6661 10902785

[34] Paulmichl K Hatunic M Højlund K Jotic A Krebs M Mitrakou A. Modification and validation of the triglyceride-to-HDL cholesterol ratio as a surrogate of insulin sensitivity in white juveniles and adults without diabetes mellitus: the single point insulin sensitivity estimator (SPISE). Clin Chem. (2016) 62(9):1211–9. 10.1373/clinchem.2016.257436. 27471037

[35] Chung WK Erion K Florez JC Hattersley AT Hivert M-F Lee CG. Precision medicine in diabetes: a consensus report from the American diabetes association (ADA) and the European association for the study of diabetes (EASD). Diabetes Care. (2020) 43(7):1617–35. 10.2337/dci20-0022 32561617 PMC7305007

