# Correction: The case for broader use of HOMA-IR in pediatric metabolic assessment: a narrative review

**DOI:** 10.3389/fped.2026.1994417

**Published:** 2026-10-23

**Authors:** Salah Snouda

**Affiliations:** Tunis University, Tunis, Tunisia

**Keywords:** childhood metabolic syndrome, HOMA-IR, insulin resistance indices, metabolic screening, pediatric insulin resistance, precision pediatric medicine, pubertal insulin resistance, targeted screening

Reference 2 was erroneously written as:

Peplies J, Jiménez-Pavón D, Savva SC, Buck C, Günther K, Fraterman A, et al. Percentiles of fasting serum insulin, glucose, HbA1c and HOMA-IR in pre-pubertal normal weight European children from the IDEFICS cohort. *Int J Obes*. (2014) **38**(9):S39–47. doi:10.1038/ijo.2014.134.

It should be:

Peplies J, Jiménez-Pavón D, Savva SC, Buck C, Günther K, Fraterman A, et al. Percentiles of fasting serum insulin, glucose, HbA1c and HOMA-IR in pre-pubertal normal weight European children from the IDEFICS cohort. *Int J Obes (Lond)*. (2014) **38**(Suppl 2):S39–47. doi:10.1038/ijo.2014.134.

Reference 3 was erroneously written as:

American Diabetes Association. 14. Children and adolescents: standards of care in diabetes—2026. *Diabetes Care*. (2025) **49**(Supplement 1):S297–314. doi:10.2337/dc26-S014.

It should be:

American Diabetes Association Professional Practice Committee for Diabetes. 14. Children and Adolescents: Standards of Care in Diabetes—2026. *Diabetes Care*. (2026) **49**(Suppl 1):S297–S320. doi:10.2337/dc26-S014

Reference 4 was erroneously written as:

Costa CS, Rauber F, Leffa PS, Sangalli CN, Campagnolo PDB, Vitolo MR. Ultra-processed food consumption and its effects on anthropometric and glucose profile: a longitudinal study during childhood. *Nutr Metab Cardiovasc Dis*. (2019) **29**:177–84. doi:10.1016/j.numecd.2018.11.003.

It should be:

Costa CS, Rauber F, Leffa PS, Sangalli CN, Campagnolo PDB, Vitolo MR. Ultra-processed food consumption and its effects on anthropometric and glucose profile: a longitudinal study during childhood. *Nutr Metab Cardiovasc Dis*. (2019) **29**(2):177–184. doi:10.1016/j.numecd.2018.11.003. PMID:30660687.

Reference 15 was erroneously written as:

Kurtoğlu S, Hatipoğlu N, Mazıcıoğlu M, Kendirci M, Keskin M, Kondolot M. Insulin resistance in obese children and adolescents: HOMA-IR cut-off levels in the prepubertal and pubertal periods. *J Clin Res Pediatr Endocrinol*. (2010) **2**(3):100–6. doi:10.4274/jcrpe.v2i3.100.

It should be:

Kurtoğlu S, Hatipoğlu N, Mazıcıoğlu M, Kendirici M, Keskin M, Kondolot M. Insulin resistance in obese children and adolescents: HOMA-IR cut-off levels in the prepubertal and pubertal periods. *J Clin Res Pediatr Endocrinol*. (2010) **2**(3):100–106. doi:10.4274/jcrpe.v2i3.100.

Reference 16 was erroneously written as:

Shashaj B, Luciano R, Contoli B, Morino GS, Spreghini MR, Rustico C, et al. Reference ranges of HOMA-IR in normal-weight and obese young Caucasians. *Acta Diabetol*. (2016) **53**(3):251–60. doi:10.1007/s00592-015-0782-4.

It should be:

Shashaj B, Luciano R, Contoli B, Morino GS, Spreghini MR, Rustico C, et al. Reference ranges of HOMA-IR in normal-weight and obese young Caucasians. *Acta Diabetol*. (2016) **53**(2):251–260. doi:10.1007/s00592-015-0782-4. PMID:26070771.

Reference 19 was erroneously written as:

Lim CYS, Foo YW, Tok CLX, Lim YY, Loke KY, Lee YS, et al. Screening for metabolic complications of childhood and adolescent obesity: a scoping review of national and international guidelines. *Obes Rev*. (2022) **23**(10):e13513. doi:10.1111/obr.13513.

It should be:

Lim CYS, Foo YW, Tok CLX, Lim YY, Loke KY, Lee YS, et al. Screening for metabolic complications of childhood and adolescent obesity: a scoping review of national and international guidelines. *Obes Rev*. (2022) **23**(12):e13513. doi:10.1111/obr.13513. PMID:36286080.

Reference 20 was erroneously written as:

Schrank Y, Biscaro A, Engiz O, Pereira AC, Krieger JE, Bloch KV, et al. *Proposal for Fasting Insulin and HOMA-IR Reference Intervals*. São Paulo: Archives of Endocrinology and Metabolism (2024).

It should be:

Schrank Y, Fontes R, Perozo AFDF, Araújo PB, Pinheiro MFMC, Gomes DMV, et al. Proposal for fasting insulin and HOMA-IR reference intervals based on an extensive Brazilian laboratory database. *Arch Endocrinol Metab*. (2024) **68**:e230483. doi:10.20945/2359-4292-2023-0483. PMID:39529982.

Reference 22 was erroneously written as:

Rodriquez IM, O’Sullivan KL. Youth-onset type 2 diabetes: burden of complications and socioeconomic cost. *Curr Diab Rep*. (2023) **23**(8):59–67. doi:10.1007/s11892-023-01501-7.

It should be:

Rodriquez IM, O’Sullivan KL. Youth-onset type 2 diabetes: burden of complications and socioeconomic cost. *Curr Diab Rep*. (2023) **23**(5):59–67. doi:10.1007/s11892-023-01501-7. PMID:36961664.

Reference 23 was erroneously written as:

Okuyan O. *New Approach to the Diagnosis of Metabolic Syndrome in Children. in Metabolic Syndrome: A Comprehensive Update*. Sharjah: Bentham Science Publishers (2025).

It should be:

Wu EL, Kazzi NG, Lee JM. Cost-effectiveness of screening strategies for identifying pediatric diabetes mellitus and dysglycemia. *JAMA Pediatr*. (2013) **167**(1):32–39. doi:10.1001/jamapediatrics.2013.419. PMID:23403824.

The originally cited source did not support the economic claim associated with Reference 23. The replacement is a pediatric screening simulation; the related text has been amended to state explicitly that the analysis did not evaluate HOMA-IR.

Reference 25 was erroneously written as:

Bauer KC, Huus KE, Brown EM, Bozorgmehr T, Woodward SE, Serapio-Palacios A, et al. Dietary Intervention Reverses Fatty Liver and Altered Gut Microbiome. *mSystems*. (2020) **5**(5):e00499–20. doi:10.1128/mSystems.00499-20.

It should be:

Goss AM, Dowla S, Pendergrass M, Ashraf A, Bolding M, Morrison S, et al. Effects of a carbohydrate-restricted diet on hepatic lipid content in adolescents with non-alcoholic fatty liver disease: a pilot, randomized trial. *Pediatr Obes*. (2020) **15**(7):e12630. doi:10.1111/ijpo.12630. PMID:32128995.

The originally cited source was a murine study and did not support the human pediatric intervention claim. The replacement is a pilot randomized trial involving adolescents with obesity and non-alcoholic fatty liver disease; the related text has been qualified accordingly.

Reference 29 was erroneously written as:

Son D-H, Lee HS, Lee Y-J, Lee J-H, Han J-H. Comparison of triglyceride-glucose index and HOMA-IR for predicting prevalence of metabolic syndrome. *Nutr Metab Cardiovasc Dis*. (2022) **32**(3):596–604. doi:10.1016/j.numecd.2021.11.017.

It should be:

Kang B, Yang Y, Lee EY, Yang HK, Kim HS, Lim SY, et al. Triglycerides/glucose index is a useful surrogate marker of insulin resistance among adolescents. *Int J Obes (Lond)*. (2017) **41**(5):789–792. doi:10.1038/ijo.2017.14. PMID:28104918.

The originally cited study involved adults and should not have been characterised as pediatric evidence. The replacement directly evaluated the TyG index in adolescents.

Reference 30 was erroneously written as:

Dundar C, Terzi O, Arslan HN. Comparison of the ability of HOMA-IR, VAI, and TyG indexes to predict metabolic syndrome in children with obesity: a cross-sectional study. *BMC Pediatr*. (2023) **23**(1):389. doi:10.1186/s12887-023-03892-8. Linked PMID:37553563.

It should be:

Dundar C, Terzi O, Arslan HN. Comparison of the ability of HOMA-IR, VAI, and TyG indexes to predict metabolic syndrome in children with obesity: a cross-sectional study. *BMC Pediatr*. (2023) **23**(1):74. doi:10.1186/s12887-023-03892-8. PMID:36765298.

Reference 31 was erroneously written as:

Hammel MC, Stein R, Kratzsch J, Vogel M, Eckert AJ, Triatin RD, et al. Fasting indices of glucose-insulin-metabolism across life span and prediction of HbA1c in children and adults. *Lancet Reg Health Eur*. (2023) **31**:100652. doi:10.1016/j.lanepe.2023.100652.

It should be:

Hammel MC, Stein R, Kratzsch J, Vogel M, Eckert AJ, Triatin RD, et al. Fasting indices of glucose-insulin-metabolism across life span and prediction of glycemic deterioration in children with obesity from new diagnostic cut-offs. *Lancet Reg Health Eur*. (2023) **30**:100652. doi:10.1016/j.lanepe.2023.100652. PMID:37465325.

Reference 32 was erroneously written as:

Sarıkaya E, Kilci F, Murat NÖ; [Author names to be confirmed upon full-text access]. Practical utility of fasting glucose, fasting insulin, HOMA-IR, HOMA-B, and HbA1c in identifying insulin resistance and dysglycemia risk in children with excess weight. *Acta Paediatr*. (2026). doi:10.1111/apa.70657.

It should be:

Sarıkaya E, Kilci F, Murat NÖ. Fasting Biomarkers and Glycated Haemoglobin Identify Children With Excess Weight at Risk of Dysglycaemia and Guide Oral Glucose Tolerance Testing. *Acta Paediatr*. (2026) 115(10):2305–14. doi:10.1111/apa.70657. PMID:42332988.

Reference 34 was erroneously written as:

Paulmichl K, Hatunic M, Engiz O, Giri D, Gasser R, Sudi K, et al. The single point insulin sensitivity estimator (SPISE) as a new tool in the diagnosis of insulin resistance in children and adolescents. *Pediatr Diabetes*. (2020) **21**(7):1089–97.

It should be:

Paulmichl K, Hatunic M, Højlund K, Jotic A, Krebs M, Mitrakou A, et al. Modification and validation of the triglyceride-to-HDL cholesterol ratio as a surrogate of insulin sensitivity in white juveniles and adults without diabetes mellitus: the Single Point Insulin Sensitivity Estimator (SPISE). *Clin Chem*. (2016) **62**(9):1211–1219. doi:10.1373/clinchem.2016.257436. PMID:27471037.

The published Reference 34 could not be verified as written. The corrected entry is the original SPISE development and validation article; the related narrative and Table 2 wording have been qualified to reflect its limited juvenile sample.

The original version of this article has been updated.

Missing/Wrong in-text citation

**Section 4.1, “The HOMA-IR formula,” final paragraph**

The final sentence was erroneously written as:

*“HOMA-IR provides a validated, cost-effective surrogate that correlates well with clamp-derived measures in pediatric populations (13).”*

It should read:

*“HOMA-IR provides a practical surrogate of basal insulin resistance (13), but it is not interchangeable with the hyperinsulinemic-euglycaemic clamp and requires age-, pubertal-stage-, population-, and assay-specific interpretation (15–20, 26, 28).”*

**Table 1**

In Table 1, in the HOMA-IR row under the “Cost” column, the text was erroneously written as:

“*Low (<$20)”*

It should read:

“*Variable by laboratory and healthcare system”*

**Section 4.4, “Comparison with alternative insulin resistance indices,” first paragraph following Table 2**

The sentence was erroneously written as:

“*The TyG index has received particular attention as a potential alternative to HOMA-IR because it circumvents the insulin assay variability problem (29).”*

It should read:

“*Compared with HOMA-IR, the TyG index has been investigated as a potential pediatric surrogate of insulin resistance because it does not require a fasting insulin measurement (29, 30).”*

**Section 4.4, “Comparison with alternative insulin resistance indices,” second paragraph following Table 2**

The sentence was erroneously written as:

“*The SPISE index, validated in European adolescent cohorts (34), offers the advantage of not requiring a fasting insulin measurement, but may be less sensitive to early-stage insulin resistance where lipid profiles remain normal.”*

It should read:

“*The SPISE index, validated in cohorts including white juveniles and adults without diabetes (34), offers the advantage of not requiring fasting insulin measurement; broader validation across pediatric populations is still needed.”*

**Section 4.5, “HOMA-IR in the context of precision pediatric medicine,” first paragraph**

Reference 35 was omitted from the reference list, and the unresolved placeholder “[ADA/EASD, 2020]” appeared in the article. The placeholder has now been replaced with the numeric citation (35), and the first sentence should read:

“*The emerging precision diabetology framework, as articulated by the ADA/EASD Consensus Report on Precision Medicine in Diabetes, emphasises that metabolic disease encompasses biologically distinct subtypes requiring individualised approaches (35).”*

Reference 35 should be added as:

Chung WK, Erion K, Florez JC, Hattersley AT, Hivert MF, Lee CG, et al. Precision Medicine in Diabetes: A Consensus Report From the American Diabetes Association (ADA) and the European Association for the Study of Diabetes (EASD). *Diabetes Care*. (2020) **43**(7):1617–1635. doi:10.2337/dci20-0022. PMID:32561617.

Reference 35 is added at the end of the reference list; the existing references retain their published numbers.

**Section 5.4, “Absence of prospective outcome data,” final paragraph**

The final two sentences were erroneously written as:

“*The arguments in this review are based on mechanistic plausibility, cross-sectional associations, and cost-effectiveness modelling, not on completed screening trials. Prospective validation remains the most urgent research priority.”*

They should read:

“*The arguments in this review are based on mechanistic plausibility and cross-sectional associations, not on completed screening trials; economic modelling specific to HOMA-IR screening is also lacking. Prospective validation remains the most urgent research priority.”*

**Section 6.1, “Direct costs of pediatric metabolic disease,” second paragraph**

The paragraph was erroneously written as:

“*Conversely, the cost of adding a fasting insulin test to a routine pediatric blood panel is typically less than $20 in most healthcare systems. A recent cost-effectiveness analysis suggested that utilising indices like HOMA-IR may allow for efficient resource allocation by identifying at-risk individuals before expensive pharmacological or surgical interventions are required (23).”*

It should read:

“*An economic simulation of pediatric diabetes and dysglycemia screening compared oral glucose tolerance, glucose-challenge, random glucose, and HbA1c strategies; it did not evaluate HOMA-IR. The cost-effectiveness of adding HOMA-IR to targeted pediatric screening therefore remains unknown (23).”*

**Section 6.3, “The window of reversibility,” first paragraph**

The second sentence was erroneously written as:

“*Intensive lifestyle and dietary interventions—particularly the restriction of ultra-processed foods and free sugars—have been shown to reduce HOMA-IR and may reverse early-stage hepatic steatosis in children and adolescents (24, 25).”*

It should read:

“*Physical activity may improve insulin resistance in youth (24). In a small pilot randomised trial involving adolescents with obesity and non-alcoholic fatty liver disease, a carbohydrate-restricted diet reduced hepatic lipid within the intervention group and produced greater reductions in HOMA-IR than a fat-restricted diet; these findings require confirmation in larger trials (25).”*

**Section 6.3, “The window of reversibility,” second paragraph**

The paragraph was erroneously written as:

“*It is important to note that this cost-effectiveness argument is primarily derived from modelling studies and extrapolation from intervention trials; direct evidence from completed population-level HOMA-IR screening programmes remains limited.”*

It should read:

“*It is important to note that direct evidence from completed population-level HOMA-IR screening programmes is limited, and the cost-effectiveness of HOMA-IR screening has not been established.”*

**Section 7.1, “Lifestyle and nutritional intervention as first-line response,” first paragraph**

The first three sentences were erroneously written as:

“*The primary and most evidence-supported response to elevated HOMA-IR in children is targeted dietary and lifestyle modification. The restriction of ultra-processed foods, free sugars, and refined carbohydrates has been shown to reduce HOMA-IR and may reverse early-stage NAFLD within weeks of implementation (24, 25). Structured physical activity—particularly resistance training and high-intensity interval training—may improve peripheral insulin sensitivity through GLUT-4 upregulation in skeletal muscle, independent of weight loss [McCormack* et al. *(24)].”*

They should read:

“*Physical activity and diet quality may improve insulin resistance in youth. In a small randomised study of adolescents with obesity, HOMA-IR decreased following an eight-week lifestyle programme, while supervised exercise improved fitness and power (24). In a separate pilot randomised trial involving adolescents with obesity and non-alcoholic fatty liver disease, a carbohydrate-restricted diet reduced hepatic lipid within the intervention group and produced greater reductions in HOMA-IR than a fat-restricted diet; these findings require confirmation in larger trials (25).”*

Table 2

In Table 2, in the SPISE row under the “What It measures” column, the text was erroneously written as:

“*Peripheral insulin sensitivity”*

It should read:

“*Whole-body insulin sensitivity (surrogate)”*

In the SPISE row under the “Advantages” column, the text was erroneously written as:

“*No fasting insulin needed; validated in adolescents”*

It should read:

“*No fasting insulin measurement needed”*

In the SPISE row under the “Limitations” column, the text was erroneously written as:

“*Requires lipid panel; less sensitive to early IR”*

It should read:

“*Requires a lipid panel; limited validation across pediatric populations”*

In the SPISE row under the “Pediatric evidence” column, the text was erroneously written as:

“*Moderate—validated in European cohorts (34)”*

It should read:

“*Initial validation included white juveniles and adults; broader pediatric validation is needed (34)”*

The original version of this article has been updated.

